# Seek, test, treat: substance-using women in the HIV treatment cascade in South Africa

**DOI:** 10.1186/s13722-017-0077-x

**Published:** 2017-04-26

**Authors:** Wendee M. Wechsberg, Charles van der Horst, Jacqueline Ndirangu, Irene A. Doherty, Tracy Kline, Felicia A. Browne, Jennifer M. Belus, Robin Nance, William A. Zule

**Affiliations:** 1 Substance Use, Gender and Applied Research, RTI International, 3040 East Cornwallis Road, Research Triangle Park, NC 27709-2194 USA; 2 Health Policy and Management, UNC Gillings School of Global Public Health, Chapel Hill, NC USA; 3 Department of Psychology, North Carolina State University, Raleigh, NC USA; 4 Psychiatry and Behavioral Sciences, Duke University School of Medicine, Durham, NC USA; 5 School of Medicine, University of North Carolina, Chapel Hill, NC USA; 6 Substance Use, Gender and Applied Research, RTI International, Pretoria, South Africa; 7 UCB Biosciences, Raleigh, NC USA; 8 Statistics and Epidemiology, RTI International, 3040 East Cornwallis Road, Research Triangle Park, NC 27709-2194 USA; 9 Psychology Department, University of North Carolina, Chapel Hill, NC USA; 10 Biostatistics, School of Public Health, University of Washington, 1410 NE Campus Parkway, Seattle, WA USA

**Keywords:** Sexually-active women, Alcohol and other drug use, Treatment cascade

## Abstract

**Background:**

Women in South Africa who use alcohol and other drugs face multiple barriers to HIV care. These barriers make it difficult for women to progress through each step in the HIV treatment cascade from diagnosis to treatment initiation and adherence. This paper examines correlates of HIV status, newly diagnosed HIV status, and use of antiretroviral therapy (ART).

**Methods:**

Outreach workers recruited sexually active Black African women who used substances in Pretoria as part of a U.S. National Institutes of Health-funded geographically clustered randomized trial examining the effect of an intervention to reduce alcohol and drug use as well as sexual risk behaviors. To address the question of interest in the current investigation, cross-sectional baseline data were used. At study enrollment, all participants (N = 641) completed an interview, and underwent rapid HIV testing and biological drug screening. Those who tested positive for HIV and were eligible for ART were asked about their barriers to initiating or adhering to ART. Bivariate and multivariable logistic regression analyses were conducted to determine correlates of HIV status, newly diagnosed HIV, and ART use.

**Results:**

At enrollment, 55% of participants tested positive for HIV, and 36% of these women were newly diagnosed. In multivariable analyses of the entire sample, women who had completed 10th grade were less likely to be living with HIV (OR 0.69; CI 0.48, 0.99) and those from the inner city were more likely to be living with HIV (OR 1.83; CI 1.26, 2.67). Among HIV-positive participants, women were less likely to be newly diagnosed if they had ever been in substance abuse treatment (OR 0.15; CI 0.03, 0.69) or used a condom at last sex (OR 0.58; CI 0.34, 0.98) and more likely to be newly diagnosed if they were physically assaulted in the past year (OR 1.97; CI 1.01, 3.84). Among women eligible for ART, fewer were likely to be on treatment (by self-report) if they had a positive urine test for opiates or cocaine (OR 0.27; CI 0.09, 0.80).

**Conclusions:**

These results, although cross-sectional, provide some guidance for provincial authorities to address barriers to HIV care for sexually active, substance-using vulnerable women in Pretoria. Targeting the inner city with prevention campaigns, expanding and improving substance abuse treatment programs, linking clients with simultaneous HIV testing and treatment, and targeting women who have experienced sexual assault and violence may help the government achieve the UNAIDS 90-90-90 treatment target.

Clinical Trials.gov NCT01497405 registered on December 1, 2011.

## Background

Despite progress in reducing HIV incidence, approximately 2 million people were newly infected worldwide in 2015 [1]. Biomedical approaches such as HIV treatment as prevention offer hope for reducing transmission even when condom use is low. In a study of HIV-discordant couples, antiretroviral therapy (ART) reduced HIV transmission by 93% [2]. The results of this study combined with findings from two ecological studies [3, 4] and several modeling studies [5] suggest that high rates of HIV testing and treatment could end the HIV epidemic. These findings led UNAIDS to establish the “90-90-90” treatment goals whereby 90% of all people infected with HIV will know their status, 90% of those diagnosed with HIV will receive ART, and 90% of those on ART will achieve undetectable HIV viral loads by 2020 [6].

However, achieving the 90-90-90 goals in key populations, such as people who use drugs, will require intensive efforts to find them, test them for HIV, link them to ART, and retain them in treatment [7]. To achieve the 90-90-90 goals in a country like South Africa, this approach—labeled “seek, test, treat, and retain” (STTR) [8]—will require expanding STTR efforts among people who use alcohol and other drugs, female sex workers, and men who have sex with men [9, 10].

With AIDS being the leading cause of death among women of childbearing age globally [11], it is critical to increase testing among women at high risk of HIV and link those who test positive for HIV to ART. Focusing STTR efforts on women in this group has the potential to reduce AIDS-related deaths among them. Treating HIV-positive women also reduces onward transmission of HIV to men who in turn transmit it to other women, which is a primary objective of STTR efforts. Moreover, treating HIV-positive women may help break the cycle in places like sub-Saharan Africa where two-thirds of new infections among 15–24-year-olds occurred among women in 2015 [1].

Alcohol and other drug use can interfere with STTR efforts at each step in the HIV treatment cascade [9]. Use of alcohol and other drugs may also reduce pre-exposure prophylaxis (PrEP) uptake and adherence [12]. Additionally, alcohol and other drug use exacerbates existing social and structural barriers to HIV testing and HIV-related services [13] and adversely affects health-seeking behavior and health care utilization [14, 15]. In South Africa where there are more people living with HIV than anywhere else in the world, alcohol and drug use is a growing problem, particularly in urban areas. Alcohol and marijuana use are most common, although the use of heroin, cocaine, Mandrax (methaqualone) and methamphetamine have increased significantly with injecting drug use also on the rise [16]. Therefore, it is especially pertinent to the South African context to examine both alcohol and specific drug use as predictors of progress through the HIV treatment cascade. In this paper, we hypothesized that recent alcohol and other drug use would be negatively associated with forward progress through the HIV treatment cascade. More specifically, we propose to determine predictors of current HIV status, newly diagnosed HIV cases and ART use. Each of these stages is important in reaching the 90-90-90 treatment goals.

## Methods

### Procedure overview

The paper presents analyses of cross-sectional baseline data collected from women who were recruited by outreach workers in Pretoria, South Africa. The complete protocol for the study was published in an open-access journal [17]. Briefly, the intervention study focuses on reducing HIV risk behaviors (e.g., alcohol or drug use, unprotected sex) in high-risk women; it also focuses on improving adherence to the Test, Treat, and Retain elements of the HIV cascade for HIV-positive women.

### Sample and eligibility

To participate in the study, women had to meet the following eligibility criteria: (1) self-identify as female; (2) be Black African; (3) be aged 15 or older (15–17-year-olds had to demonstrate tacit emancipation, which shows their capacity as a minor to act without parental consent^1^) [18]; (4) report using alcohol or other drugs at least weekly during the past 90 days; (5) report unprotected vaginal sex with a male partner in the past 6 months; (6) speak English, Sesotho, Tswana, or Zulu; (7) consent to HIV rapid testing (blood), drug testing (urine), and alcohol breathalyzer; (8) provide written and verbal assent/consent to participate; and (9) provide verifiable locator information for the Pretoria area and plan to stay in the area for the next 12 months.

### Recruitment

A total of 641 women enrolled in the study between May 2012 and September 2014. All of the outreach workers for the study were trained on community outreach techniques for approaching women in public places and discreetly screening them for eligibility. Outreach workers received additional training on procedures specific to this study such as how to schedule appointments and transport women safely to the field site for enrollment and data collection. Given the differences in living conditions, access to drugs, and population density across the greater Pretoria area, we differentiated where women were recruited—either within the inner city (i.e., the Central Business District) or outside the inner city. Women recruited from the inner-city often spent time in transient forms of shelter or workplace establishments such as abandoned houses or street corners.

Outreach workers conducted eligibility screening and scheduled intake appointments for those who were eligible and interested. The appointments, conducted at the study’s field site, included the following activities: rescreening for eligibility, informed consent, a computer-assisted personal interview, a finger stick of blood for rapid HIV testing, collection of a urine sample for pregnancy testing and drug screening, and a breathalyzer test for recent alcohol use. Participants received toiletries worth R100 (at the time of the study, $1 US equaled approximately 10 South African Rand) for their time and reimbursement for travel.

### Self-report measures

The primary data collection instrument—the Pretoria Risk Behavior Assessment (PRBA)—included sections on sociodemographic characteristics, alcohol and other drug use, substance abuse treatment, sexual behavior, violence perpetration and victimization, and physical and mental health. In a previous study in Pretoria, the PRBA demonstrated high reliability and validity [19].

#### Sex risk

Three measures of sex risk were included: the number of sex acts (anal and vaginal) without a condom in the past 30 days; condom use at last sex; and engaging in sex work or trading sex for drugs, money, food, clothing, shelter, or any other goods in the past 6 months.

#### Alcohol and other drug use

Measures included use of marijuana, cocaine (includes powder cocaine and crack), heroin by itself, and Nyaope (heroin mixed with marijuana). For each substance, participants were asked if they had ever used it (i.e., lifetime use). Participants who reported lifetime use were asked the number of days they had used the substance in the past 30 days. We recoded these variables into any use of each drug in the past 30 days. We used the Alcohol Use Disorders Identification Test (AUDIT) to assess problem drinking using a cut-point of 20, which indicates probable alcohol dependence and a need for alcohol treatment [20].

#### Victimization

The PRBA included the following ordered items to measure physical and sexual abuse:

- Has anyone ever physically hurt you (i.e., someone hurt you by striking or beating you to the point that you had bruises, cuts, or broken bones)?
  - When was the last time you were physically hurt?
- Has anyone ever pressured you or forced you to participate in sexual acts against your will?
  - When was the last time you were pressured or forced to participate in sexual acts against your will?

The analyses used derived variables for physical abuse and for sexual abuse in the past year.

#### Mental and physical health

We used the Mental Health scale from Version 2 of the SF-36 Health Survey [21] to assess participants’ mental health and the Vitality scale to assess physical health. Raw scores were computed and transformed according to instructions in the manual. Specifically, scale scores were transformed using the following formula: [(Actual raw score − lowest possible raw score)/(possible raw score range)] × 100. Higher scores on the Mental Health and Vitality scales are associated with better functioning. Our focus in this paper was to identify factors that distinguish among women in the sample at each step in the HIV treatment cascade. Therefore, we did not compare SF-36 scale scores in the sample to scores in the broader population in South Africa.

#### Antiretroviral therapy (ART)

To assess current ART status, the questionnaire asked participants who reported ever testing positive for HIV the following questions: “Have you ever taken any ARV medications? Are you taking any ARV medications now?”

#### Barriers to HIV treatment initiation and retention

We assessed barriers to HIV treatment initiation, retention in treatment, and adherence to HIV medication with the question: “Why have you stopped or not taken ARV (*anti*-*HIV*) medications, either now or in the past?” A list of response options followed the question along with instructions to “check all that apply.” Participants also had the option to respond “other” and specify an unlisted response. The research team collapsed the responses into the themes.

### Biological tests

#### Alcohol and other drug use

A breathalyzer was used to assess recent alcohol use. Participants provided a urine sample for drug testing. A multipanel drug test was used to assess marijuana (THC), cocaine, ecstasy (MDMA), and opiate use (Homemed Multi drug 5-Panel Dipcard).

#### HIV status

All participants were tested for HIV at baseline regardless of self-report. Testing was done by collecting a blood sample from a finger stick and performing two parallel rapid HIV tests (First Response^®^ HIV 1-2.0 or ABON^®^-Alere HIV 1/2/O Tri-line HIV Rapid Test and iCARE One Step HIV [1&2] Whole Blood/Serum/Plasma Test). If one test was positive and the other test was either indeterminate or negative, then a confirmatory test was performed (Alere Determine™ HIV-1/2). A participant was classified as HIV positive if any two of the tests were reactive.

#### Housing

We captured differences in level of disadvantage by asking participants whether they lived in an informal settlement. In South Africa, most informal settlements are made up of a collection of shacks that have not been formally approved by the government. Some informal settlements may exist for years and have hundreds of thousands of residents.

#### Recruitment area

Women were differentiated based on location of recruitment because the city of Pretoria consists of varying levels of disparity. Women were classified as having been recruited from the inner city versus outside the inner city. The inner city indicates that women were recruited from within the Central Business District and often were found in transient forms of shelter or near drug-dealing establishments such as abandoned houses or high drug traffic street corners.

#### Historical factors

The recruitment period for this study overlapped with the South African government’s campaign to increase HIV testing and treatment. Therefore, we included month of recruitment in our models to assess the campaign’s success in reaching our population and as a potential confounder.

### Statistical analysis

We used multivariable logistic regression analyses to test hypotheses regarding associations between independent variables of interest (e.g., alcohol, drug use, sex work) and HIV status, newly diagnosed with HIV, and being on ART. The analysis examining correlates of HIV status included all women in the study (n = 641). The analysis examining correlates of newly diagnosed HIV included all HIV-positive women (n = 354) whereas the analysis examining factors associated with ART use included only HIV-positive women who were eligible for ART (n = 156). Bivariate analyses examined the associations between dependent variables and each covariate. Covariates that were statistically significant at *p* < .05 in bivariate analysis were included in the multivariable model. When two highly correlated independent variables were both significantly associated with an outcome in bivariate analyses, we included only one of them in the multivariable model based on substantive and theoretical considerations. In the ART model, we excluded one variable (ever in substance abuse treatment) from the multivariable model because of small cell sizes. Analyses were performed in SPSS version 23 (IBM Corp.; Armonk, NY).

## Results

Of the 1012 women screened for eligibility, 772 (76%) met all of the eligibility criteria. Most (86%) of the women who were ineligible did not meet the criterion of self-reported alcohol or other drug use at least once a week over the previous 90 days. Among eligible women, 83% (641/772) enrolled into the study. Women who declined to participate and those who enrolled into the study were similar with respect to age, language, and having a main sex partner; 53% of women who declined to participate reported sex work compared with 43% of women who enrolled in the study (*p* = .01).

Only three women reported ever injecting illicit drugs. Self-reported use of heroin—either alone or mixed with marijuana (Nyaope)—and biological test results for opiates were similar (17 vs. 18%, *p* > .05). Slightly more women reported marijuana use in the past 30 days than had a positive drug screen result (38 vs. 31%, *p* > .05). Significantly fewer women reported using cocaine in the past 30 days than tested positive for it (12 vs. 14%, *p* < .05). However, despite the statistical significance, even these results were quite similar.

### HIV testing and prevalence (“seek and test”)

**Fig. 1:**
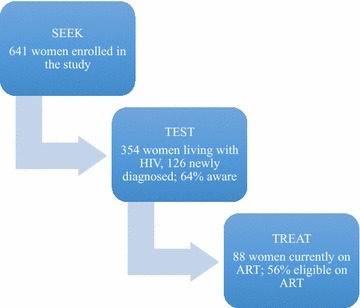
HIV cascade for women recruited for the study

During their baseline interview, 90% of the participants reported having been tested for HIV previously. At enrollment in the study, 55% (n = 354) of women had a positive test result for HIV. Table 1 displays characteristics of the participants stratified by HIV status as well as results from the bivariate and multivariable analyses. In Fig. 1, we present the study participants reached along the treatment cascade of seek, test and treat being only baseline data. In multivariable analyses, being older, engaging in sex work, being recruited in the inner city, and living in an informal settlement were associated with increased odds of testing positive for HIV, whereas more education had a protective effect.

**Table 1:** Correlates of HIV among high-risk women in Pretoria, South Africa

Variable	HIV status		Bivariate logistic regression	Multivariable logistic regression
	Negative	Positive		
Sociodemographic characteristic	(n = 287)	(n = 354)	OR (95% CI)	OR (95% CI)
*Age in years, mean (SD)*	29.0 (8.5)	31.5 (7.0)	1.04 (1.02, 1.07)***	1.03 (1.01, 1.05)**
% education ≥10th grade	70.4	56.5	0.55 (0.39, 0.76)***	0.69 (0.48, 0.98)*
% unemployed	82.7	87.7	1.49 (0.93, 2.38)	
% married or living as married	46.7	52.5	1.26 (0.93, 1.73)	
% ever had a family member with HIV/AIDS	59.1	64.9	1.28 (0.93, 1.76)	
% ever incarcerated (lifetime)	29.7	39.5	1.55 (1.11, 2.15)**	1.10 (0.76, 1.59)
% ever in substance abuse treatment (lifetime)	7.0	5.6	0.80 (0.42, 1.52)	
*Urine drug screen and breathalyzer results*				
% marijuana (THC) positive	31.4	31.4	1.0 (0.71, 1.40)	
% opiate positive	19.2	16.9	0.86 (0.57, 1.29)	
% cocaine positive	15.7	13.3	0.82 (0.53, 1.28)	
% opiate or cocaine positive	21.3	20.3	0.95 (0.64, 1.39)	
% alcohol (breathalyzer) positive	12.9	15.3	1.22 (0.77, 1.91)	
Mean breathalyzer score for women with a positive alcohol test (SD)	0.08 (0.11)	0.08 (0.11)	1.69 (0.03, 82.96)	
*Self*-*report alcohol and drug use past 30* *days*				
% alcohol	84.7	88.7	1.42 (0.90, 2.25)	
% marijuana	28.6	30.5	1.01 (0.78, 1.55)	
% cocaine (rock)	13.2	10.5	0.76 (0.47, 1.24)	
% heroin and marijuana mixed (Nyaope)	18.8	15.3	0.78 (0.51, 1.17)	
Self-report Alcohol Use Disorders Identification Tests (AUDIT) score ≥20	26.5	34.2	1.44 (1.02, 2.03)*	1.43 (0.99, 2.07)
*Sex risk behaviors*				
% used a condom at last sex	28.5	43.3	1.92 (1.37, 2.68)***	1.45 (0.97, 2.16)
# times unprotected sex past 30 days, mean (SD)	8.6 (12.9)	8.9 (14.2)	1.00 (0.99, 1.01)	
Sex work past 6 months	26.8	45.2	2.25 (1.61, 3.14)***	1.47 (0.98, 2.22)
*Victimization*				
% physically assaulted past year	9.1	13.3	1.54 (0.93, 2.55)	
% sexually assaulted past year	5.2	7.3	1.44 (0.75, 2.77)	
*SF*-*36 mental health and vitality scales*				
Mental health scale score, mean (SD)^a^	65.1 (19.2)	64.3 (19)	1.00 (0.99, 1.01)	
Vitality scale score, mean (SD)^a^	34.2 (18.3)	35.7 (17.2)	1.00 (1.00, 1.01)	
*Contextual factors*				
% recruited from inner city^b^	24.4	40.4	2.1 (1.49, 2.96)***	1.90 (1.30, 2.78)**
Century month recruited^c^, mean (SD)	150.7 (8.5)	150.3 (8.7)	0.99 (0.98, 1.01)	
% living in informal settlement^d^	30.3	38.4	1.43 (1.03, 2.00)*	1.49 (1.04, 2.14)*

* *p* < .05; ** *p* < .01; *** *p* < .001

^a^SF-36 Mental Health and Vitality scales

Transformed scale = [(Actual raw score − lowest possible raw score)/(possible raw score range)] × 100

^b^The reference group is recruited from outside the inner city

^c^Century month (CM). January 2001 = 1. The project recruited from May 2012 (CM = 136) to September 2014 (CM = 164). South Africa was in the midst of scaling up HIV testing and treatment during this period

^d^The reference group is not living in an informal settlement

### Newly diagnosed with HIV

Among the 354 women who tested positive for HIV, 36% (n = 126) were newly diagnosed. Of these, 27% reported never receiving an HIV test. Among those who had been tested, the median number of months since their most recent HIV test was 23. Table 2 displays the results related to being newly diagnosed with HIV. More specifically, the multivariable analysis shows that being older, having a history of substance abuse treatment, and using a condom at last sex were associated with decreased odds of being newly diagnosed with HIV, whereas physical assault in the past 12 months was associated with increased odds of being newly diagnosed.

**Table 2:** Correlates of newly diagnosed HIV among HIV-positive women in Pretoria, South Africa

Variable	Previously diagnosed	Newly diagnosed	Bivariate logistic regression	Multivariable logistic regression
Sociodemographic characteristic	n = 228	n = 126	OR (95% CI)	OR (95% CI)
*Age in years, mean (SD)*	32.7 (6.9)	29.1 (7.0)	0.92 (0.89, 0.95)***	0.91 (0.88, 0.95)***
% education ≥10th grade	57.5	54.8	1.11 (0.71, 1.75)	
% unemployed	89.1	85.3	0.71 (0.36, 1.41)	
% married or living as married	54.4	49.2	0.81 (0.53, 1.26)	
% ever had a family member with HIV/AIDS	66.1	62.7	0.86 (0.55, 1.36)	
% ever incarcerated (lifetime)	40.8	37.3	0.86 (0.55, 1.35)	
% ever in substance abuse treatment (lifetime)	7.9	1.6	0.19 (0.04, 0.82)*	0.15 (0.03, 0.69)*
*Urine drug screen and breathalyzer results*				
% marijuana (THC) positive	33.3	27.8	0.77 (0.48, 1.24)	
% opiate positive	17.1	16.7	0.97 (0.54, 1.73)	
% cocaine positive	14.0	11.9	0.83 (0.43, 1.6)	
% opiate or cocaine positive	20.2	20.6	1.03 (0.60, 1.76)	
% alcohol (breathalyzer) positive	16.3	13.5	0.80 (0.43, 1.49)	
*Sex risk behaviors*				
% used a condom at last sex	48.9	33.3	0.52 (0.33, 0.82)**	0.58 (0.34, 0.98)**
# times unprotected sex past 30 days, mean (SD)	8.2 (12.9)	10.2 (16.3)	1.01 (0.99, 1.02)	
% engaged in sex work past 6 months	50.0	36.5	0.58 (0.37, 0.90)*	0.83 (0.49, 1.40)
*Victimization*				
% physically assaulted past year	10.5	18.3	1.90 (1.02, 3.52)*	1.97 (1.01, 3.84)*
% sexually assaulted past year	6.6	8.7	1.36 (0.60, 3.05)	
*SF*-*36 mental health and vitality scales*				
Mental health scale score, mean (SD)^a^	58.4 (14.4)	59.0 (15.8)	1.00 (0.99, 1.02)	
Vitality scale score, mean (SD)^b^	36.6 (16.9)	33.9 (17.7)	0.99 (0.98, 1.00)	
*Contextual factors*				
% recruited from inner city^b^	40.4	40.5	1.01 (0.65, 1.57)	
Century month recruited^c^, mean (SD)	150.3 (8.9)	150.3 (8.4)	1.00 (0.97, 1.02)	
% living in informal settlement^d^	36.4	42.1	1.27 (0.81, 1.98)	

* *p* < .05; ** *p* < .01; *** *p* < .001

^a^SF-36 Mental Health and Vitality scales

Transformed scale = [(Actual raw score − lowest possible raw score)/(possible raw score range)] × 100

^b^The reference group is recruited from outside the inner city

^c^Century month (CM). January 2001 = 1. The project recruited from May 2012 (CM = 136) to September 2014 (CM = 164). South Africa was in the midst of scaling up HIV testing and treatment during this period

^d^The reference group is not living in an informal settlement

### Treat

Among the 228 women who reported during their baseline interview that they knew they were living with HIV, 72 of these women reported that they were not eligible for ART because their CD4 count was too high. Of the 156 women who were eligible for ART, 88 (56%) reported being on ART. The analyses of factors associated with being on ART exclude the 72 women who were not eligible for it (see Table 3). In multivariable analyses, older age and later month enrolled in the project were significantly associated with increased odds of being on ART, whereas having a positive urine drug screen for either opiates or cocaine metabolites was associated with significantly decreased odds of being on ART. In additional analyses to clarify the association between a history of substance abuse treatment and not being on ART, we found that 80% of women with a history of substance abuse treatment tested positive for opiates or cocaine.

**Table 3:** Factors associated with ART use among women who knew they were living with HIV and were eligible for ART

Variable	Not on ART	Currently on ART	Bivariate logistic regression	Multivariable logistic regression
Sociodemographic characteristic	n = 68	n = 88	OR (95% CI)	OR (95% CI)
*Age in years, mean (SD)*	30.7 (7.0)	35.9 (6.5)	1.13 (1.07, 1.19)***	1.08 (1.02, 1.15)**
% education ≥10th grade	60.3	53.4	0.92 (0.48, 1.78)	
% unemployed	86.0	86.7	1.07 (0.38, 2.96)	
% married or living as married	52.9	60.2	1.35 (0.71, 2.55)	
% family member with HIV/AIDS	59.7	79.5	2.62 (1.29, 5.35)**	2.09 (0.90, 4.82)
% ever incarcerated (lifetime)	51.5	28.4	0.37 (0.19, 0.73)**	
% ever in substance abuse treatment (lifetime)	16.2	1.1	0.06 (0.01, 0.47)**	
*Urine drug screen and breathalyzer results*				
% marijuana (THC) positive	50.0	19.3	0.24 (0.12, 0.49)***	
% opiate positive	30.9	4.5	0.11 (0.03, 0.33)***	
% cocaine positive	25.0	4.5	0.14 (0.05, 0.45)**	
% opiate or cocaine positive	35.3	6.8	0.13 (0.05, 0.35)***	0.27 (0.09, 0.80)*
% alcohol (breathalyzer) positive	14.7	15.9	1.10 (0.45, 2.65)	
*Sex risk behaviors*				
% used a condom at last sex	49.2	39.8	0.68 (0.36, 1.30)	
# times unprotected sex past 30 days, mean (SD)	10.3 (18.7)	7.5 (9.4)	0.99 (0.96, 1.01)	
% engaged in sex work past 6 months	58.8	38.6	0.44 (0.23, 0.84)*	
*Victimization*				
% physically assaulted past year	13.2	8.0	0.57 (0.20, 1.61)	
% sexually assaulted past year	11.8	4.5	0.36 (0.10, 1.24)	
*SF-36 mental health and vitality scales*				
Mental health scale score, mean (SD)^a^	56.1 (13.5)	60.6 (14.0)	1.02 (1.00, 1.05)*	1.02 (1.00, 1.04)
Vitality scale score, mean (SD)^b^	42.2 (18.8)	33.4 (14.4)	0.97 (0.95, 0.99)*	
*Contextual factors*				
% recruited from inner city^b^	54.4	26.1	0.30 (0.15, 0.58)***	
Century month recruited^c^, mean (SD)	147.0 (8.8)	153.6 (8.3)	1.09 (1.05, 1.13)***	1.06 (1.01, 1.11)*
% living in informal settlement^d^	32.4	38.6	1.32 (0.68, 2.56)	

* *p* < .05; ** *p* < .01; *** *p* < .001

^a^SF-36 Mental Health and Vitality scales

Transformed scale = [(Actual raw score − lowest possible raw score)/(possible raw score range)] × 100

^b^The reference group is recruited from outside the inner city

^c^Century month (CM). January 2001 = 1. The project recruited from May 2012 (CM = 136) to September 2014 (CM = 164). South Africa was in the midst of scaling up HIV testing and treatment during this period

^d^The reference group is not living in an informal settlement

### Barriers to treatment initiation and retention

When this study began recruiting, South Africa offered ART only to patients whose CD4 cell count was below 350 cells/ml. By the time recruitment ended, the government had raised the threshold for starting ART to 500 cells/ml. Tying eligibility for ART to CD4 cell count means many women were not eligible for ART.

Among participants who were eligible for ART, the primary barriers to ART initiation and retention were structural factors such as the clinic not providing ART, and individual factors, such as missing appointments (Table 4). Personal beliefs and perceived need for ART remain problematic.

**Table 4:** Barriers to obtaining or adhering to ART

Theme	Specific items^a^	N	%
Missed appointments	Missed staging appointment, did not attend wellness appointment	24	35.3
Structural factors	Change in clinic, clinic not providing ART, lack of transportation, no identification card, no food, no place to store pills	23	33.8
Personal beliefs about ART and own readiness	Fear of side effects, fear to commit to daily dose, no need for ART, not feeling “ready,” too much trouble to pick up	16	23.5
In the process of obtaining ART	Pending CD4 results, need to schedule appointment, currently attending wellness appointment	10	14.7
Other medical issues and difficulties with dosage	Only took when pregnant, getting tuberculosis treatment, need alcohol and other drug treatment first, did not follow dosage, kept missing dosage	7	10.3
Interpersonal influence	Stigma, not disclosed HIV status, boyfriend	5	7.4

^a^Not mutually exclusive

## Discussion

With 55% of the sample testing positive for HIV, this study demonstrates the feasibility of using community outreach to find (*seek*) women at high risk for HIV and *test* them. Women’s biological susceptibility [22] combined with social determinants such as economic dependence and inequitable gender norms contribute to the high prevalence and incidence of HIV among women in developing countries [23, 24]. To prevent new infections among women in these settings, STTR efforts will need to include the men who are infecting women in addition to women. However, the most direct way to prevent women in these settings from acquiring HIV may be to offer them PrEP [25]. Contrary to our hypothesis, there was no relationship between testing positive for alcohol or other drugs and testing positive for HIV in this sample. This may be due the fact that all women in the study had to report use of alcohol and other drugs.

Similar to HIV status, there was no association between recent drug and alcohol use and being newly diagnosed with HIV in this sample, which may be explained by the high rates of HIV testing in the sample. Although 90% of the entire sample and 95% of women who tested positive for HIV reported a previous HIV test, 36% of women with a positive test were unaware of their status. Among the 92 newly diagnosed women who reported a previous HIV test, the median number of months since their most recent test was 23. To achieve the goal of 90% of HIV-positive women in this population knowing their HIV status, more women need to be tested, and they must be tested more frequently—preferably at least once a year and more frequently, if symptomatic. Because almost all of these women likely acquired HIV through sex with men, increases in HIV testing and treatment among women must be accompanied by similar increases among men to reduce incidence. Unfortunately, efforts to test and treat Black African men in South Africa have been less successful [26, 27].

During their baseline interview, only 56% of the 156 women who were aware of their HIV status and eligible for ART reported receiving it. In the multivariable model, the significant association between being recruited later in the project and being on ART suggests that the South African government’s efforts to increase HIV testing and treatment are beginning to work. Nevertheless, much more work remains to be done before South Africa will achieve the 90-90-90 goals.

In contrast to the other steps in the HIV cascade, recent drug use was associated with not being on ART among women who already knew they were living with HIV. Women who had a urine drug screen result positive for cocaine or opiate metabolites were only about 25% as likely to report being on ART compared with women who tested negative. This finding is consistent with previous studies that reported heavy drug use interfered with ART initiation and retention [28]. In addition, the association between being older and being on ART may be due to older women who have been infected longer becoming symptomatic, thus leading them to seek treatment.

Findings from this paper highlight the urgent need for additional resources to increase access to treatment for substance use disorders. Studies have shown that integrating the continuum of care with substance use and HIV treatment programs can decrease alcohol and drug use and offer better overall health outcomes among patients living with HIV [29, 30]. Providing greater access to treatment for substance use disorders and co-locating health departments that offer ART and substance use treatment has the potential to increase ART initiation and retention.

In January 2015, South Africa began implementing the 2013 World Health Organization recommendation to start ART at a CD4 count of 500 instead of 350 [31]. While this change removes a barrier to ART for women with CD4 counts between 350 and 500, as of 2016, South Africa had not committed sufficient resources to implement the recommendation fully. The individual and structural barriers to ART reported in this study were similar to those reported in previous studies [32] that have interfered with achieving the 90-90-90 goals. Although the new WHO guidelines recommends providing ART to everyone regardless of CD4, and the South Africa Minister of Health announced support for these recommendations, each Province in South Africa will have to come to terms with on the ground barriers to meeting these needs [33].

Our results could be used to inform future efforts to reduce barriers to testing as well as efforts to increase treatment access and adherence to achieve the 90-90-90 target. The independent association between older age and HIV infection highlights the importance of offering prevention interventions, particularly when testing younger women. Given some of the staffing constraints in health clinics, efforts in Pretoria should focus on the inner city because women recruited from the inner city were nearly twice as likely to test positive for HIV as women recruited from other areas of Pretoria. Clearly, women who have been sexually assaulted or experienced violence should routinely be offered HIV testing. Offering substance abuse treatment at the same site where ART is offered could increase adherence to ART and help address the complex issues of substance use. Free transportation to clinics or mobile treatment vans with directly observed therapy would help as well. The positive associations between being engaged in sex work and being recruited from the inner city or living in an informal settlement provide additional support for testing sex workers and women in both contexts who use alcohol or other drugs.

## Limitations

As with all cross-sectional studies, caution should be used in drawing causal inferences from associations in the multivariable models. Additionally, the study relies primarily on self-report data, which is susceptible to recall errors and socially desirable responses that may reduce the reliability and validity of the results. However, the original Risk Behavior Assessment demonstrated adequate reliability and validity [34]. Additionally, interviewers underwent intensive training and monitoring to minimize eliciting socially desirable responses and used calendars to assist participants with recall. Caution should be used in extrapolating these findings to other populations and to women in other geographic locations.

## Conclusions

South Africa faces enormous challenges to achieving the UNAIDS 90-90-90 goals for HIV testing, treatment, and viral suppression. The country will need to expand the scope and increase the frequency of HIV testing among sex workers and other women who use alcohol or other drugs.

The finding that HIV-positive women recruited later in the study were more likely to be on ART than women recruited earlier suggests that South Africa is making progress in scaling up ART. Nevertheless, only 44% of women who met the current eligibility criteria for ART self-reported they were on it, and that figure drops to 30% under the new World Health Organization guidelines to treat everyone regardless of their CD4 count. South Africa faces enormous challenges just to treat 90% of people who are aware of their HIV status. The high HIV incidence rates among women suggest that for HIV treatment as prevention to be successful, STTR efforts will need to be scaled up among the men who are infecting them as well as among women.

## Abbreviations

ART: antiretroviral therapy
ARV: antiretroviral
CI: confidence interval
CM: century month
HIV: human immunodeficiency virus
IRB: Institutional Review Board
MDMA: 3,4-methylenedioxy-methamphetamine
OR: odds ratio
PRBA: Pretoria Risk Behavior Assessment
PrEP: pre-exposure prophylaxis
SD: standard deviation
STTR: seek, test, treat, and retain
THC: tetrahydrocannabinol

## Footnotes

1 According to South African law, adolescents under 18, the age of majority, can provide consent unassisted as long as they can provide evidence of tacit emancipation. Tacit emancipation occurs when the capacity of a minor to act without parental consent is 'enlarged' to encompass certain key areas that will enable him or her to be viewed by the law as a major. The prime consideration is the degree of financial independence that the minor has achieved. In this respect, ownership of a business or an occupation that brings in a salary is key. Residence outside the parental home is regarded as further proof of emancipation.
